# Exergetic Analysis, Optimization and Comparison of LNG Cold Exergy Recovery Systems for Transportation

**DOI:** 10.3390/e20010059

**Published:** 2018-01-13

**Authors:** Paweł Dorosz, Paweł Wojcieszak, Ziemowit Malecha

**Affiliations:** Department of Cryogenic, Aeronautic and Process Engineering, Wroclaw University of Science and Technology, 50-370 Wrocław, Poland

**Keywords:** LNG, cryogenic power cycles, exergetic analysis, LNG fueled vehicles

## Abstract

LNG (Liquefied Natural Gas) shares in the global energy market is steadily increasing. One possible application of LNG is as a fuel for transportation. Stricter air pollution regulations and emission controls have made the natural gas a promising alternative to liquid petroleum fuels, especially in the case of heavy transport. However, in most LNG-fueled vehicles, the physical exergy of LNG is destroyed in the regasification process. This paper investigates possible LNG exergy recovery systems for transportation. The analyses focus on “cold energy” recovery systems as the enthalpy of LNG, which may be used as cooling power in air conditioning or refrigeration. Moreover, four exergy recovery systems that use LNG as a low temperature heat sink to produce electric power are analyzed. This includes single-stage and two-stage direct expansion systems, an ORC (Organic Rankine Cycle) system, and a combined system (ORC + direct expansion). The optimization of the above-mentioned LNG power cycles and exergy analyses are also discussed, with the identification of exergy loss in all components. The analyzed systems achieved exergetic efficiencies in the range of 20% to 36%, which corresponds to a net work in the range of 214 to 380 kJ/kg${}_{LNG}$.

## 1. Introduction

Natural gas (NG) is recognized as the cleanest available fossil fuel. The combustion of NG results in a much lower emission of pollutants compared to any other tyep of fuel (coal, lignite, and crude oil-based fuels). In liquid state (*LNG—Liquefied Natural Gas*), its volume is about 600 times lower than it is in gaseous state. This makes storage much more effective and permits long-distance transportation.

The shares of NG and LNG in the global energy market are predicted to further increase in the future [1,2]. The demand for a reduction in greenhouse gas emissions continues to generate growing interest in the potential usage of LNG as a possible fuel for transportation. Especially in the case of road transport for heavy vehicles, where diesel fuel may be replaced by LNG [3,4].

Another important application for LNG includes marine transportation. The International Marine Organization has established Emission Controlled Areas (ECA) where the emission of nitrogen oxides, sulphur oxides, and particulates must be reduced. In July 2017, the Marine Environment Protection Committee 71 adopted amendments to regulation 13 of the MARPOL (the International Convention for the Prevention of Pollution from Ships) Annex VI, introducing new limits on NOx emissions from ship exhausts for the ECA area, including the Baltic Sea and the North Sea [5]. These emissions can be substantially reduced by using LNG, which is recognized as an environmentally friendly and ecological fuel for both present and future methods of ship propulsion [6,7,8].

The last stage of the LNG technological cycle (either in LNG import terminals or the fuel system of LNG fueled vehicles) is vaporization and a temperature increase to ambient. Conventional vaporizers mainly use ambient environments as heat sources. However, there are also vaporizers which use available waste heat or heat from natural gas combustion as heat sources. Additionally, in the case of vehicle fuel systems, plate heat exchangers (PHE) can be incorporated as vaporizers instead of shell and tube vaporizers, as they provide high-intensity heat exchange and therefore require less volume. The presented research focuses on the possibility of recovering exergy from transportation systems. Exergy is the amount of useful work that can be obtained from a system by bringing it to thermodynamic equilibrium with its surroundings [9]. LNG is a cryogenic fluid and, therefore, has high exergy density. Exergy analysis is also a very convenient method of designing and improving energy systems, and has seen an increase in application in recent years [10,11]. In its physical state, it is very different than the physical state of its surroundings because the temperature of LNG is much lower than ambient.

In the case of conventional vaporizers, the exergy contained in LNG is destroyed. Many authors have reported the possibility of utilizing LNG cold exergy [12,13,14]. LNG can be a source of cooling power [15,16,17]. The cold energy from LNG regasification can be used in the air separation process [18], the freeze desalination process [19], or to improve the capacity of Adsorbed Natural Gas (ANG) tanks [1]. LNG can be also used as a heat sink in a power cycle using ambient or waste heat as the heat source. The most popular cold power cycles are: the direct expansion cycle [20], the Rankine cycle [21,22,23], the Brayton cycle [24], the absorption power cycle [25], the Stirling cycle [26,27,28] and combined cycles [29,30,31,32]. In most existing cryogenic plants, the recovery method for LNG exergy is based on direct expansion and ORC cycles [14]. The analyses performed in the presented research will focus on these methods as the most popular, and also with the greatest potential for application in transportation systems.

## 2. LNG Physical Exergy Recovery

LNG production requires a lot of energy, first to cool down the gas and then to liquefy it. The minimal work required to liquefy the NG is approximately 900 kJ/kg [33]. However, the efficiencies of real systems are considerably below ideal levels, and real liquefaction work is in the range of 2500–3500 kJ/kg, depending on the system configuration [34,35]. Only part of the energy used to liquefy natural gas is accumulated as LNG physical exergy and can be recovered during regasification, while the rest is lost due to irreversibilities in the liquefaction process. The physical exergy of LNG can be obtained using Equation (1).

(1) $$e_{LNG}=h\left(T,p\right)-h_{0}\left(T_{0},p_{0}\right)-T_{0}\left[s\left(T,p\right)-s_{0}\left(T_{0},p_{0}\right)\right]$$

The composition of LNG is not constant and can vary depending on the supplier. In the presented research, the assumed composition corresponds to the requirements of a light LNG [36] for the Polish LNG terminal and is presented in Table 1.

The physical exergy of LNG in the proposed composition is equal to 1040 kJ/kg. Taking into account the mass flow of the LNG that is potentially needed in heavy transport systems, the possible energy recovery seems to be relatively high. The current analysis focuses on LNG exergy recovery in transport systems which use LNG as a fuel.

### 2.1. Cooling Power Production in the LNG Regasification Process

**Figure 1:**
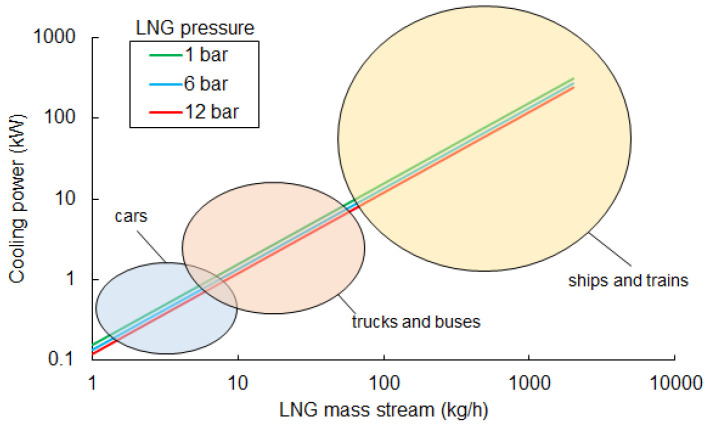
Recoverable cooling power from LNG, depending on the mass stream and pressure in the storage tank.

The simplest way to utilize waste cold available from LNG vaporization is to use it as a source of cooling power in air conditioning, refrigeration, or technological purposes. This kind of solution does not significantly complicate the LNG regasification system. However, it is limited by the demand for cooling power. The potential cooling power that may be recovered in transport systems is presented in Figure 1. The values of the LNG mass stream in the vehicles were calculated based on data regarding engine power consumption [6,7,37,38]. The maximum available cooling power was determined using the following equation:

(2) $${\dot{Q}}_{cooling}\left(p\right)={\dot{m}}_{LNG}\left(h\left(T_{0},p\right)-h_{sat.liq.}\left(p\right)\right)$$

where: $T_{0}$—ambient temperature, $h_{sat.liq.}$—specific enthalpy of saturated liquid.

Cars with an average LNG consumption rate in the range of a few kg/h are observed to have less than 1 kW of available cooling power, regardless of pressure. This value is lower than the demand for cooling power in most cars, which is about 2–3 kW. In this case, the only way to recover waste cold is by using it to support car air conditioning systems, but due to low values of available cooling power, these systems cannot be economically justified. A waste cold recovery system may be considered in trucks and buses as well. The cooling power that may be recovered is in an order of kWs and may be used to support air conditioning systems or cooling systems in refrigerated trailers. In the case of heavy transport, available cooling power is significantly higher. The highest values can be reached in marine vessels. The engine power of the vessels fed by NG is in the range of 2–12 MW. Consumption of NG, which has a calorific value greater than 28 MJ/Nm${}^{3}$, ranges from 650 to 3700 Nm${}^{3}$/h. LNG consumption ranges from 0.8 to 4.7 m${}^{3}$/h, respectively. Consequently, the mass stream of LNG is in an order of hundreds of kg/h. Possible cooling power is in the order of tens of kW, even up to hundreds of kW for large vessels. This amount of cooling power can be used in air conditioning or on-board cold rooms (e.g., cooling or freezing chambers).

Cooling power recovery systems should always be preceded by thermal and economic analyses. The systems discussed above are the simplest. However, they are not justified in the absence of a demand for cooling power. For more general purposes, LNG exergy should be utilized to generate mechanical work or electricity. This can be achieved by using a direct expansion system, the ORC (Organic Rankine Cycle) system, or a combination of both.

### 2.2. Process Modelling and Assumptions

The authors have analyzed 3 thermodynamic cycles used for power generation that utilize LNG physical energy: the direct expansion cycle, the ORC, and a coupled direct expansion—ORC cycle. The thermodynamic simulation was performed with Aspen HYSYS V10 (36.0.0.249) software. The list of all assumed simulation parameters (taken from the available literature as an average of the values published in papers [21,39,40]) is provided in Table 2. The considered systems were assumed to have an ambient heat source. All the expanders in the considered systems were fed with regasified LNG (or another regasified working fluid in the case of the ORC cycle), heated to a temperature of 283 K. Moreover, the authors assumed that liquid fraction does not occur in the expanders and that the system must be free of insufficient underpressure.

The energy needed to pump the heating medium in the evaporators of the considered systems and their efficiency was omitted for more clarity in the discussion. This energy is less than 1% of the energy required for the ORC pump, and can be neglected. Moreover, a similarly-sized pump is also present in traditional regasification systems. Exergetic efficiency was calculated for each system as useful exergy extracted from the system into its input exergy, using the following formula [41]:

(3) $$\eta_{ex}=\frac{w_{net}}{e_{in}}$$

### 2.3. Direct Expansion System

**Figure 2:**
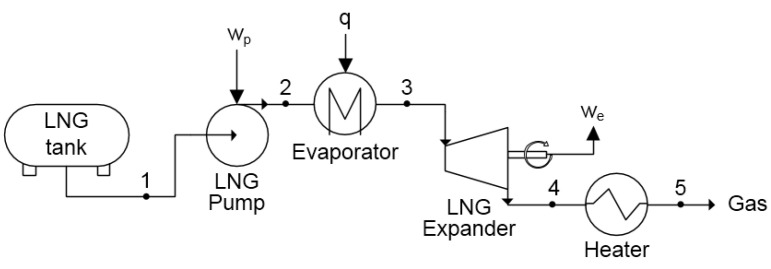
Scheme of the direct expansion system.

In the case of a direct expansion system, LNG is used as a working fluid in the cycle to produce electricity. A scheme of the system is presented in Figure 2. LNG pressure is increased in the pump (1–2), which is required to provide work ($w_{p}$). Next, the LNG is evaporated and heated to a temperature close to 283 K (point 3). The compressed gas is then expanded to ambient pressure (point 4) and expansion work $w_{e}$ is generated. In the end, the gas is heated in the heat exchanger to ambient temperature (4–5).

The exergy of LNG in the tank is equal to the exergy $e_{1}$ in point 1:

(4) $$e_{1}=\left(h_{1}-h_{0}\right)-T_{0}\left(s_{1}-s_{0}\right)$$

where $h_{0}$ and $s_{0}$ are the enthalpy and entropy of LNG in ambient conditions, respectively.

**Figure 3:**
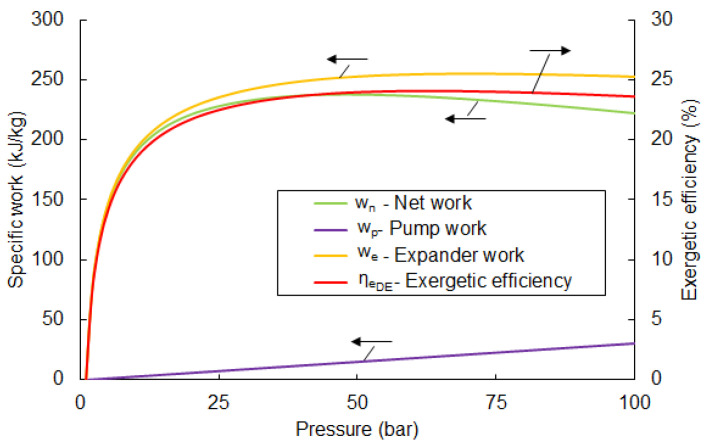
The exergetic efficiency of the direct expansion system depends on pumping pressure. The black arrows, associated with each curve, points to the corresponding vertical axis.

The exergetic efficiency $\eta_{eDE}$ of the direct expansion system is derived from (3):

(5) $$\eta_{exDE}=\frac{w_{e}-w_{p}}{e_{1}}$$

where $w_{p}$—specific work of the pump, $w_{e}$—specific work of the turbine. Based on the above equations, calculated values of the exergetic efficiency in the function of the pumping pressure are given in Figure 3.

From Figure 3, the highest possible value of $\eta_{exDE}$ is 24.1% and observed at a pumping pressure of 63 bar. A further increase in pumping pressure causes a drop in the efficiency value. Parameters for each point for the optimal pumping pressure are listed in Table 3.

The available net work, $w_{net}=w_{e}-w_{p}$ for the mentioned parameters is 236 kJ/kg. The value of efficiency rises significantly in the pressure range of 1 to 25 bar. A further increase in pressure causes only slight changes.

An important issue in the optimization of the discussed system is determining which component is responsible for the main loss of exergy. Exergy loss in the LNG pump $\Delta e_{p}$ can be calculated using:

(6) $$\Delta e_{p}=\left(e_{1}-e_{2}\right)-w_{p}$$

where the exergy of LNG in point 2 was calculated analogously to Equation (4).

Exergy loss in the evaporator $\Delta e_{evap}$ can be determined by the following formula:

(7) $$\Delta e_{evap}=e_{2}-e_{3}$$

Finally, exergy loss in the expander is:

(8) $$\Delta e_{e}=\left(e_{3}-e_{4}\right)-w_{e}$$

The temperature of the gas after expansion is lower than ambient. In the heater, exergy of the cold gas is destroyed. Therefore, exergy loss can be expressed as an external loss which equals exergy of the gas in point 4 (see Figure 2):

(9) $$\Delta e_{4}=e_{4}=\left(h_{4}-h_{0}\right)-T_{0}\left(s_{4}-s_{0}\right)$$

**Figure 4:**
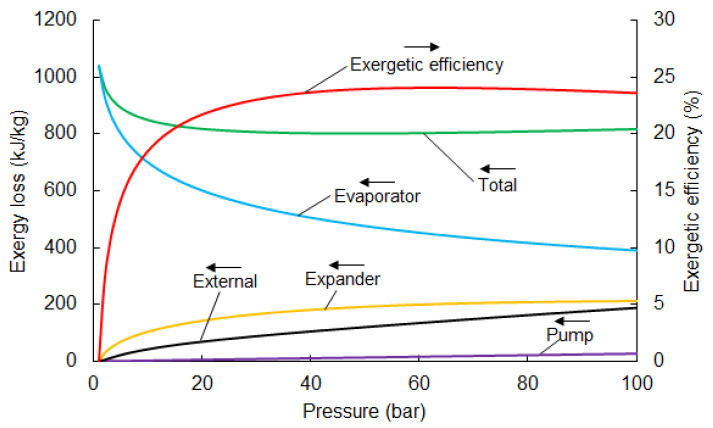
Exergy loss in the components of the direct expansion system depending on pumping pressure.

Figure 4 shows the individual losses in the components. The main loss of exergy occurs in the evaporator. The recovery of evaporation exergy is not considered in direct expansion systems and is destroyed. This is responsible for approximately 50% of the total loss (in the case of an optimal pumping pressure of 67 bar). Direct expansion cycles only use the pressure-related part of LNG physical exergy, while thermal exergy is destroyed in the heat exchange process.

**Figure 5:**
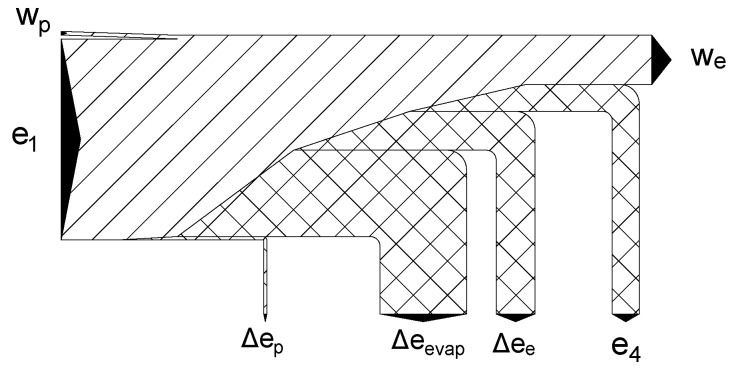
Exergy flowchart for the direct expansion cycle at an optimal pressure of 67 bar.

The temperature behind the expander declines when pumping pressure increases and, consequently, the external loss increases. The loss in the expander is comparable with the external loss, and is about 20% of the total loss. Minor losses occur in the pump and are only responsible for a small percentage of total loss. The distribution of losses for an optimal pumping pressure of 67 bar is presented in Figure 5.

### 2.4. Two-Stage Direct Expansion Cycle

To increase turbine power in the direct expansion system, multiple expansion stages can be considered. This requires an additional heat exchanger to heat the expanded gas before it goes to the next stage. Maximum work is reached when work generated by individual expanders are equal.

**Figure 6:**
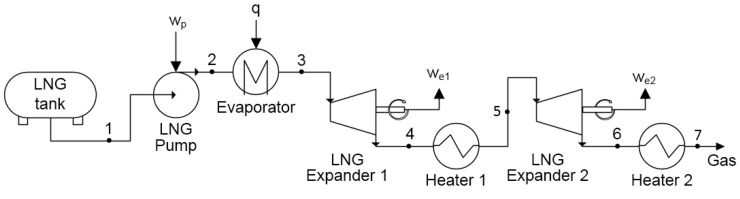
The scheme of the two-stage direct expansion exergy recovery system.

The following analysis focuses on a two-stage system, as shown in Figure 6. To optimize the working parameters, intermediate pressure was calculated using the following formula:

(10) $$p_{int}=\sqrt{p_{l}\cdot p_{h}}$$

where $p_{l}$ is the pressure of the gas (behind the expanders) and $p_{h}$ is pumping pressure.

The exergy efficiency of a system like this can be defined as the ratio of net work to LNG exergy:

(11) $$\eta_{ex_{DE2st}}=\frac{w_{e1}+w_{e2}-w_{p}}{e_{1}}$$

**Figure 7:**
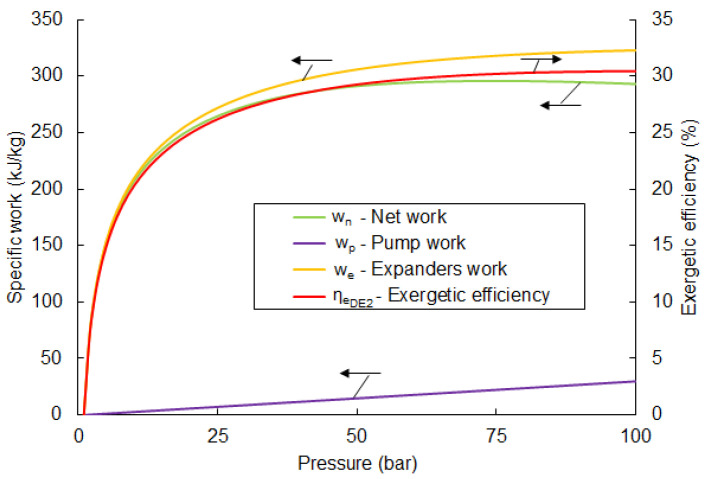
Exergetic efficiency of the two-stage direct expansion exergy recovery system.

The results of the exergetic efficiency of the two-stage direct expansion cycle are shown in Figure 7. The highest value of efficiency is observed with a pumping pressure of 100 bar at 30.4%. Exceeding this value for pressure causes liquid fraction to occur in the expander. Therefore, an analysis for higher pressures is not justified. In comparison with the single-stage direct expansion system, efficiency can be higher by, about, 20%.

**Figure 8:**
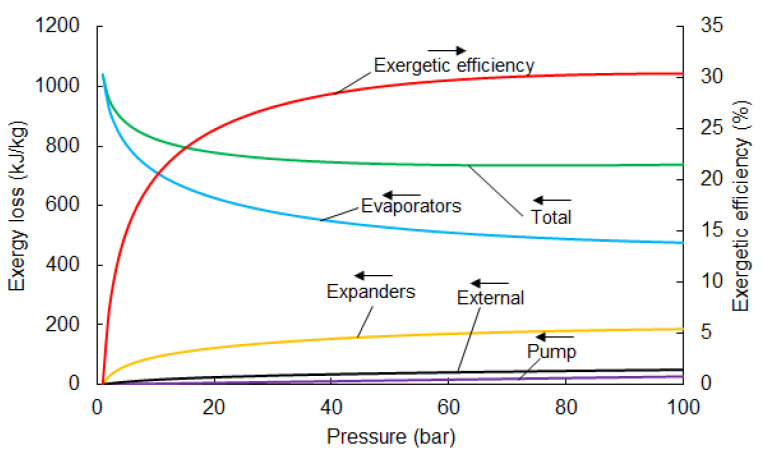
Analysis of losses in the components of the two-stage direct expansion exergy recovery system.

**Figure 9:**
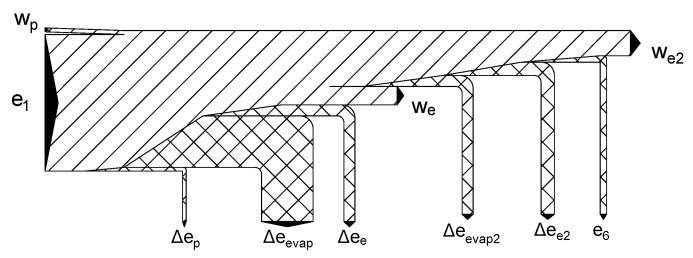
Exergy flowchart of the two-stage direct expansion cycle for an optimal pumping pressure of 100 bar.

The analysis of losses in the components of the two stage system was analogous to the singlestage direct expansion system. Internal loss in the pump, evaporators, and turbines was calculated by Equations (6)–(8) respectively. External loss is equal to the value of exergy in point 6 (see Figure 7) and was calculated using Equation (9). Exergy loss in individual components are depicted in Figure 8 and the exergy flowchart for optimal pumping pressure is presented in Figure 9. The parameters for each point of optimal pump pressure are shown in Table 4.

The two-stage expansion system helps decrease external loss and loss in the evaporators, as opposed to the single-stage expansion system. However, it is both more complicated and expensive and this should be considered in economic and risk analyses.

### 2.5. The Organic Rankine Cycle System

**Figure 10:**
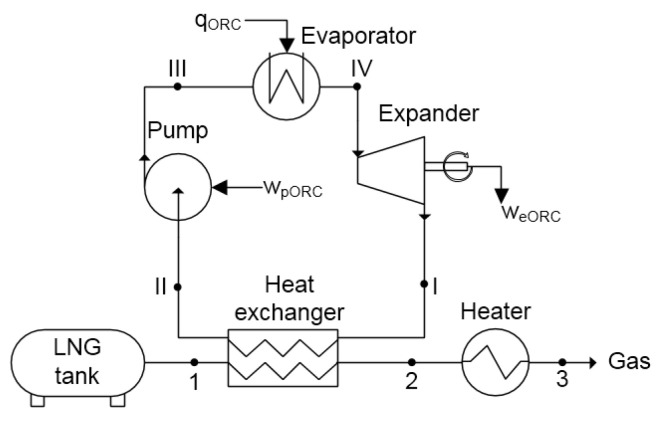
Scheme of the LNG exergy recovery system using the Organic Rankine Cycle.

The Organic Rankine Cycle can be used to recover LNG exergy if the latent heat of LNG is used as a low temperature source in the ORC cycle. A scheme of this system is depicted in Figure 10. LNG from the tank goes to the heat exchanger, where it evaporates (1–2). Then, in gas form, it goes to the heater and is heated to a temperature close to ambient.

In the ORC system, low pressure working fluid is condensed in the heat exchanger (I–II). Then, the pressure of the working liquid is increased in the pump (II–III). The liquid is evaporated in the evaporator as a result of heat exchange with air or sea water (III–IV). Next, the pressurized gas is expanded in the turbine (IV–I), where work $w_{e}$ is generated. The expanded gas returns to the heat exchanger and the cycle is closed.

The main issue in ORC systems is with selecting an optimal working fluid. The ORC evaporator was assumed to be fed with a temperature of 283 K. It can be ambient air or sea water or any other available heat source depending on the particular application, e.g., engine cooling liquid largely available on marine vessels.

The majority of ORC systems use heat sources with much higher temperatures, which influences the selection of the working fluid. As previously mentioned, slight overpressure behind the expander (point I) must be maintained. This is important, due to the fact that most components are not designed to work in conditions of insufficient pressure. Moreover, liquid fraction at the outlet of the expander cannot occur.

Recalling the assumptions given in Table 2, five working fluids have been considered and are listed in Table 5. The most important characteristic of the working fluid is a low freezing point. In order to avoid condensation pressures below atmospheric pressure, another very important factor is normal boiling temperature. To maintain a large difference between evaporation and condensation temperatures, the working fluid boiling temperature should be close as possible to the LNG temperature in the heat exchanger. On the other hand, condensation temperatures below a normal boiling point implies that there is insufficient pressure in the system.

Following the main motivation for LNG fueled vehicles related to the ecological aspects of the LNG systems, the analyzed fluids were chosen to minimize any harmful impact on the ozone layer and greenhouse effect. For all fluids, the ODP (Ozone Depletion Potential) is equal to 0. The GWP (Global Warming Potential) factor for the gases from the hydrocarbon group is relatively low, whereas for HFC-23 and PFC-14, it is much higher. However, these fluids are approved for use in low-temperature installations.

The exergetic analysis was performed for all of the working fluids from Table 5. In the case of the system with the ORC cycle, the exergetic efficiency was calculated using equation:

(12) $$\eta_{ex_{ORC}}=\frac{{\dot{m}}_{ORC}\left(w_{e_{ORC}}-w_{p_{ORC}}\right)}{{\dot{m}}_{LNG}\cdot e_{1}}$$

**Figure 11:**
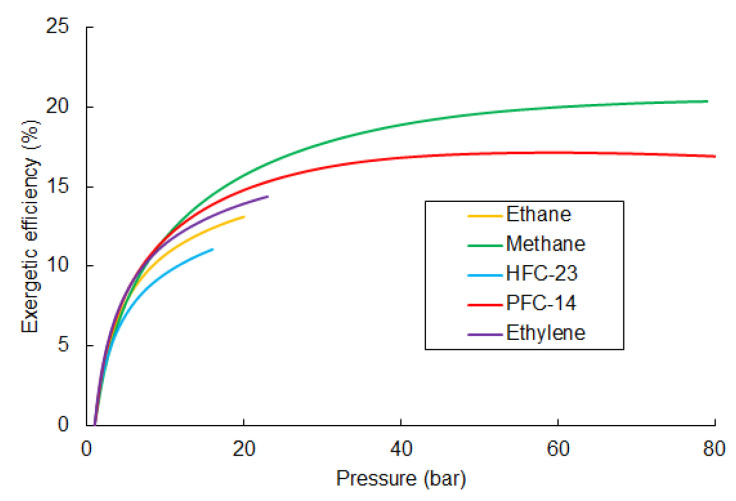
Exergetic efficiency of the ORC exergy recovery system for the considered working fluids as a function of the pumping pressure.

The changes in exergy efficiency $\eta_{ex_{ORC}}$ in the function of the pumping pressure for different working fluids are shown in Figure 11. In these calculations, the pressure of LNG was assumed to be 1 bar.

The highest efficiency was observed for methane, at close to 20%. The maximum value was reached with the highest pumping pressure of 79 bar. Comparable results were obtained for ethane, but with less efficiency—around 17%. Due to the fact that liquid fraction occurs in the expander, other fluids cannot exceed around 20 bar of pumping pressure. Parameters at each point of optimal methane ORC cycle shown in Table 6.

As in the previous cases, an analysis of exergy loss in individual components of the ORC system was performed. The internal exergy loss was calculated analogously to the direct expansion system, so loss in the pump, evaporator, and expander could be calculated using Equations (6)–(8) respectively. Exergy loss in the heat exchanger was determined by the following formula:

(13) $$\Delta e_{HX}=\left(e_{1}-e_{2}\right)-\frac{{\dot{m}}_{ORC}}{{\dot{m}}_{LNG}}\left(e_{I}-e_{II}\right)$$

The external loss is equal to exergy in point 2, since exergy in the heater is destroyed (process 2–3 in Figure 10):

(14) $$\Delta e_{2}=e_{2}=\left(h_{2}-h_{0}\right)-T_{0}\left(s_{2}-s_{0}\right)$$

**Figure 12:**
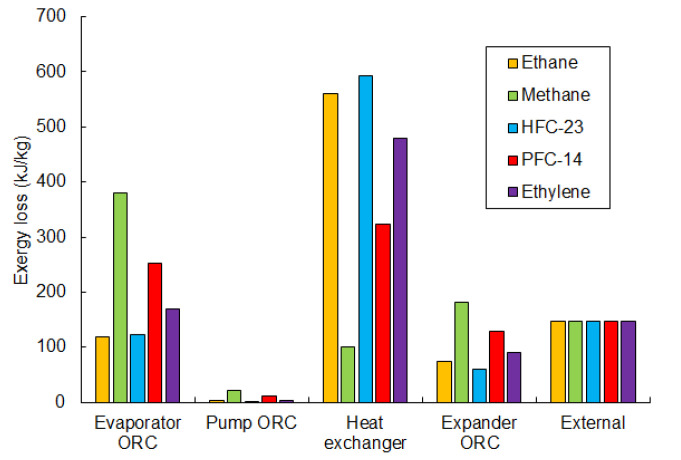
Comparison of the individual exergy losses in the components of the ORC exergy recovery system for the optimal pumping pressure and for every considered working fluid in the ORC cycle.

**Figure 13:**
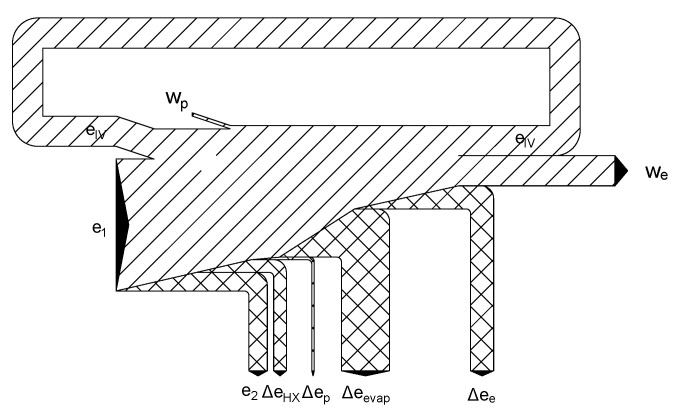
Exergy flowchart of the ORC system using methane as a working fluid for optimal pressure 79 bar.

Figure 12 summarizes and compares the losses in the individual components of the considered exergy recovery system, depending on the working fluid of the ORC cycle. The comparison is done for optimal pumping pressure (the highest efficiency). Major loss is seen to occur in the heat exchangers (evaporator and heat exchanger) and exceeds 55% for all working fluids. The losses in the heat exchanger and evaporator depend on the temperature difference between the fluids. A high difference causes large losses, as seen in the examples of methane and ethane. On the one hand, the temperature difference between ethane and LNG in the heat exchanger is high, which causes the greatest loss. On the other hand, for the same fluid, the loss in the evaporator is the lowest, due to the low temperature difference between ambient and ethane. Figure 13 presents the exergy flowchart for ORC cycle using methane as the working fluid.

The external loss is similar for every considered working fluid (about 146 kJ/kg), because the physical exergy of LNG in point 2 is the same for all of the analyzed cases.

The loss in the expander is related to the inlet pressure and is the greatest for methane and PFC-14, at 182 kJ/kg and 129 kJ/kg respectively. Minor losses are generated in the pump, and are less than 2% of the total loss for all working fluids.

### 2.6. Combined System: Direct Expansion and ORC

**Figure 14:**
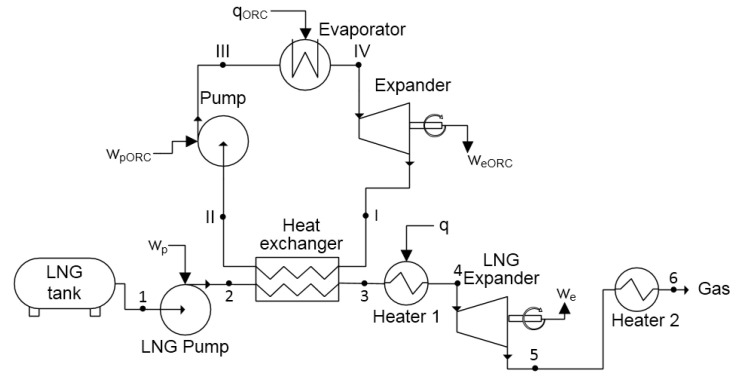
Scheme of the combined exergy recovery system consisting of the direct expansion and the ORC cycle.

A system which uses both the direct expansion and ORC cycle is called a combined system. This solution is the most complicated. However, the highest amount of exergy can be recovered. A scheme of this system is presented in Figure 14. LNG pressure is increased in the LNG pump (1–2), and then the LNG is evaporated in the heat exchanger (2–3). Next, the compressed gas is heated to ambient temperature (3–4) and then goes to the expander where it is expanded (4–5) and work ($w_{e}$) is generated. After this, low pressure gas is heated (5–6) to ambient temperature in heater 2. Simultaneously, the low pressure gas in the ORC cycle goes to the heat exchanger where it is liquefied by the heat exchanged with the LNG stream (I–II). Next, the pressure of the ORC working fluid is increased in the pump (II–III) and evaporated, and then heated to ambient temperature in the evaporator (III–IV). Consequently, the high pressure gas is expanded (IV–I) and work $w_{e_{ORC}}$ is generated. From the expander, low pressure gas returns to the heat exchanger and the cycle is closed.

In the combined system, an important issue is with selecting an appropriate working fluid for the ORC cycle. Due to the fact that LNG pressure is increased, methane cannot be used in the ORC cycle, since the pressure behind the turbine would have to be greater than the LNG pressure. For further analysis, four fluids were selected: ethane, propane, HFC-23, and PFC-14. The exergetic efficiency of the combined system using these fluids was calculated with Equation (15). In this case, the efficiency can be defined as follows:

(15) $$\eta_{ex_{com}}=\frac{\frac{{\dot{m}}_{ORC}}{{\dot{m}}_{LNG}}\left(w_{e_{ORC}}-w_{p_{ORC}}\right)+w_{e}-w_{p}}{e_{1}}$$

**Figure 15:**
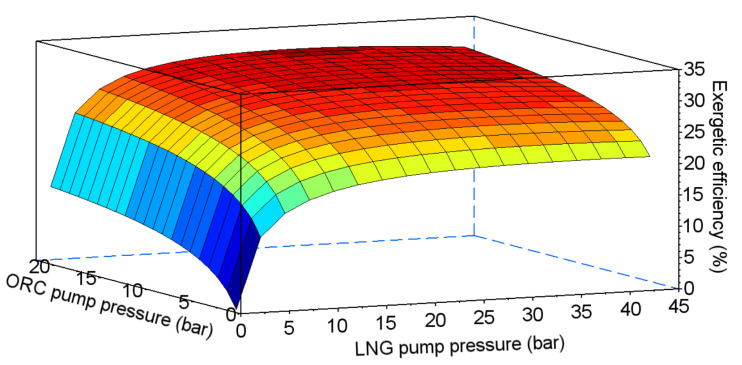
Exergetic efficiency for the combined system using ethane as the working fluid for the ORC cycle.

**Figure 16:**
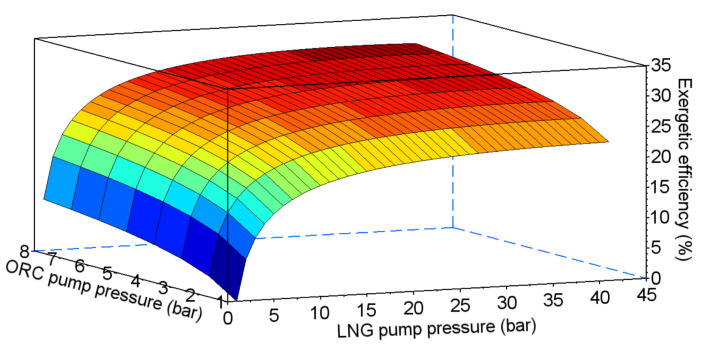
Exergetic efficiency for the combined system using propane as the working fluid for the ORC cycle.

**Figure 17:**
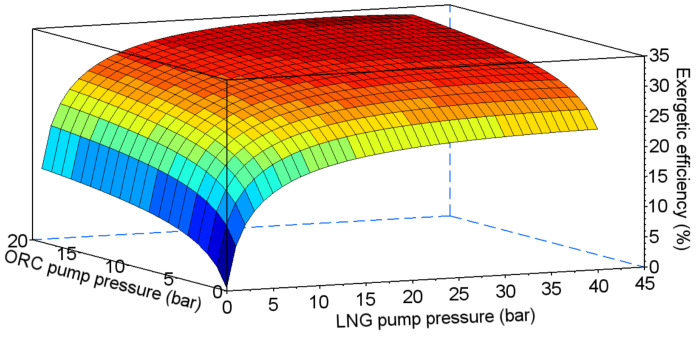
Exergetic efficiency for the combined system using HFC-23 as the working fluid for the ORC cycle.

**Figure 18:**
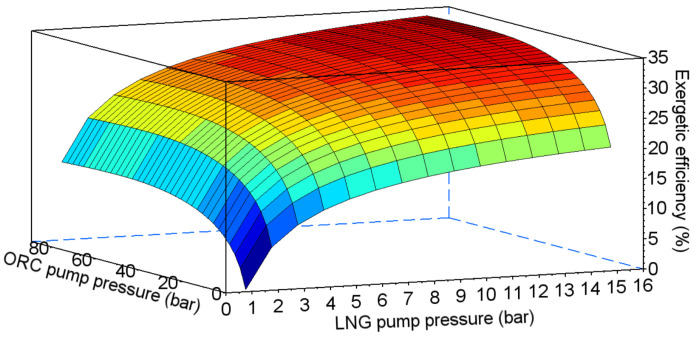
Exergetic efficiency for the combined system using PFC-14 as the working fluid for the ORC cycle.

In the combined system, efficiency depends on two pumping pressures—in the ORC cycle and in the direct expansion system (points III and 2 in Figure 14 respectively). The results of the exergetic efficiency analysis performed for ethane, propane, HFC-23, and PFC-14 are presented in Figure 15, Figure 16, Figure 17 and Figure 18 respectively.

The values of the optimal pumping pressures and the maximum exergetic efficiency are presented in Table 7. The highest value of exergetic efficiency was reached with ethane, at 36.2%. The efficiencies for HFC-23 and PFC-14 were lower by, about, 5%. The lowest value of efficiency was obtained for propane, at 30%.

To investigate the exergy losses for the individual components of the system, a similar analysis was performed. Exergy loss in the LNG and ORC pumps were calculated by Equation (6), in the LNG and ORC evaporator by Equation (7), in the LNG and ORC expanders by Equation (8), and in the heat exchanger by Equation (13). External exergy loss (exergy in point 5 in Figure 14) was calculated by by Equation (14).

**Figure 19:**
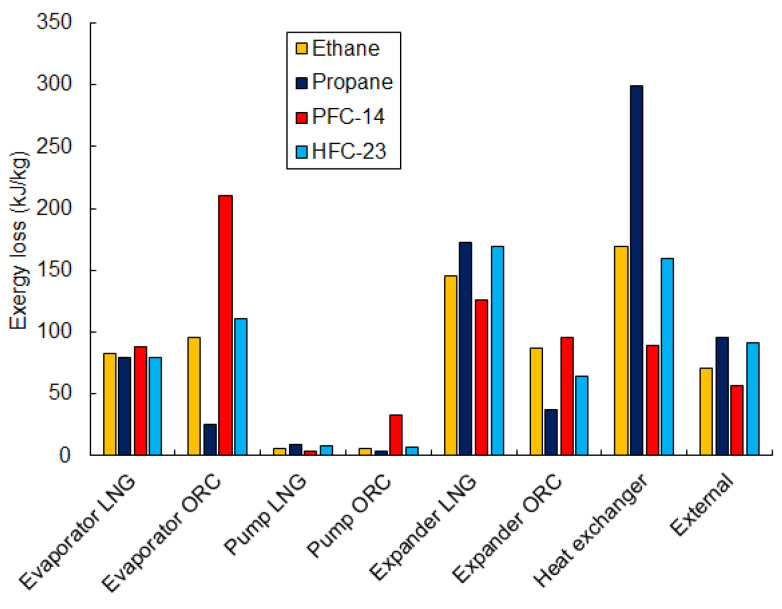
Exergy losses in the components of the combined exergy recovery system for the considered working fluids of the ORC cycle.

Exergy losses for different working fluids are presented in Figure 19. The losses for ethane can be seen as the most optimized. It is worth noting that the smallest losses in the LNG and ORC evaporators are for propane. However, for the same fluid, loss in the heat exchanger is much larger than for any other working fluid. The smallest loss in the heat exchanger is obtained with PFC-14, but the losses in the ORC and LNG evaporators are also highest. The losses in the expanders are relatively high and are responsible for, about, 30% of total loss. Lower losses occur in the pumps and are below 5%. Considering that only latent LNG exergy is used in the heat exchanger, the value of external loss is, about, 10% of the total loss and equal to 71 kJ/kg for ethane. Due to the fact that the losses for ethane are the most balanced, this makes it the most optimal working fluid and ensures the highest efficiency of exergy recovery.

**Figure 20:**
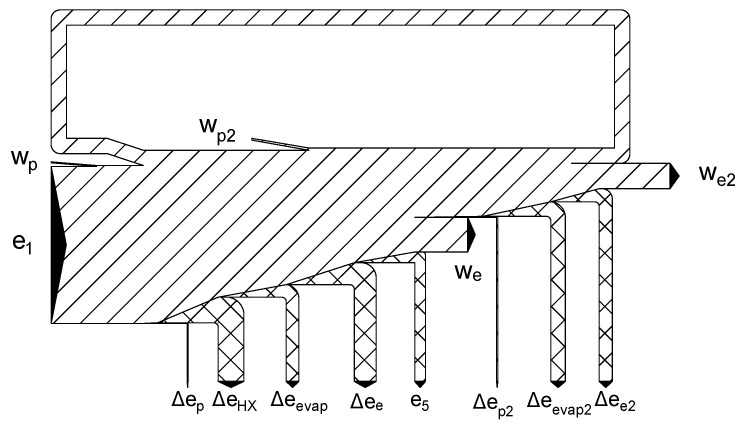
Exergy flowchart of the combined system using ethane as the working fluid in the ORC cycle for an optimal LNG pumping pressure of 21 bar and ORC pumping pressure of 36 bar.

The exergy flow chart for ethane with optimal pumping pressures (ORC pressure: 36 bar and LNG pressure: 21 bar) is presented in Figure 20. The list of all the parameters for that cycle is shown in Table 8.

## 3. Conclusions

**Figure 21:**
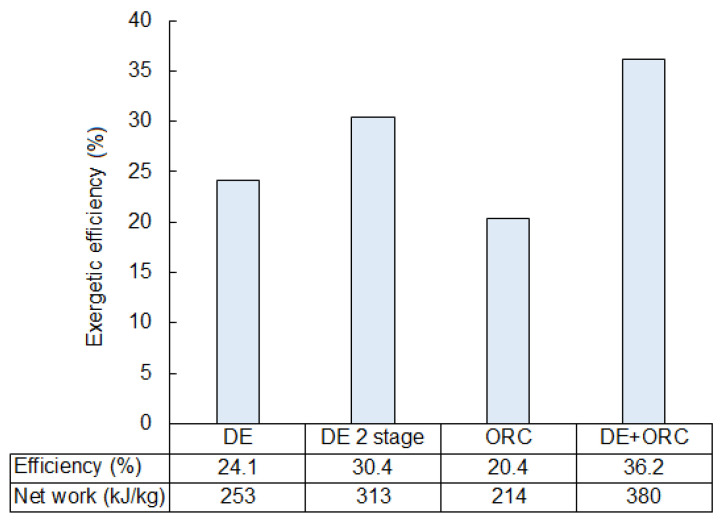
A comparison of exergetic efficiencies for the considered exergy recovery systems.

The considered exergy recovery systems are able to recover 20 to 36% of LNG exergy. A comparison of efficiency for the analyzed solutions is provided in Figure 21. The ORC system is observed to be the least efficient −20% of which corresponds to 214 kJ/kg. Nevertheless, the main advantage of this system is that LNG is not being expanded as in the direct expansion systems. The fuel supply system is separated from components with moving parts (pumps, turbines, etc.) and is more reliable.

The ORC system can be applied as well in a situation when LNG cannot be pumped and then expanded to low pressure, e.g., in the case of vehicles with high pressure gas engines where the required natural gas pressure at the engine inlet can be as high as 300 bar. The ORC recovery system can be further optimized by choosing the appropriate working fluid and optimizing the ORC pumping pressure.

The direct expansion recovery system and the two-stage direct expansion system are able to recover 24% and 30% of LNG exergy, respectively. These methodologies can only be applied if LNG can be used as a working fluid. In addition, in this case, they are the simplest methods available to recover exergy for producing electricity. The disadvantage of these types of systems is that heat from vaporization is wasted and released into the external surroundings. Nevertheless, efficiency is still higher compared to the ORC recovery system.

For the parameters assumed in the presented analyses, the two-stage system is able to recover up to 313 kJ/kg, and the one-stage system, up to 253 kJ/kg. However, an additional turbine and evaporator increases the investment costs and results in a decline in reliability. The selection of an appropriate system must be preceded by economic and risk analyses.

The highest exergetic efficiency was achieved with the combined system, which connects the direct expansion and ORC systems. The value of efficiency was, about, 36%, which corresponds to 380 kJ/kg of expander work. However, this is a compound system and requires more space. This could be a very important factor if the recovery system were to be dedicated for use in transportation, where free space may be strongly limited. Moreover, a large number of components can make this system less reliable in comparison with simpler systems.

Knowing the values for exergetic efficiency and the specific work make it possible to obtain the electric power of the considered systems. However, the presented study can be treated as a more general study as it can be utilized beyond transportation. Figure 21 shows an estimate of the possible exergy recovery efficiency, and this can be used as a general reference for any other application where the discussed cycles can be used.

The presented study was conducted for a specific composition of LNG, as seen in Table 1. Changes to the composition would only slightly affect the temperature of LNG evaporation and the maximum allowable pressure in a direct expansion cycle. Consequently, the results obtained for a different composition of LNG could only slightly vary from the results presented in this paper. The proposed systems could be successfully utilized for different compositions of LNG as well.

The greatest possible amount of electric power is produced by the system with the highest exergetic efficiency. For small vessels, where LNG consumption is around 1000 kg/h, the corresponding recovered electric power starts from 60 kW for the ORC system, and reaches up to 105 kW for the combined system. The production of electricity from the exergy recovery systems grows linearly with LNG consumption. For larger vessels, LNG consumption may reach 2000 kg/h, which corresponds to 211 kW of electricity. The recovered electricity can noticeably reduce the operating costs of the LNG powered vessel. The amount of electric power produced is not justified for cars, trucks and buses. Due to relatively low LNG consumption, the amount of electric power that may be retrieved is in the order of single kWs. Other limitations may be related to available space. Moreover, in road transportation, the demand for electric power is not significant. Hence, a better option is to recover LNG waste cold for air conditioning or cooling systems as proposed in Section 2.1.

## Figures and Tables

**Table 1:** Qualitative requirements for light LNG at Świnoujście terminal [36].

Parameter	Value
CH${}_{4}$ content	95.4% (mol)
C${}_{2}$H${}_{6}$ content	3.2% (mol)
N${}_{2}$ content	1.4% (mol)
Density at *T* = 113.35 K	442 kg/m${}^{3}$
Molar mass	16.66 g/mol

**Table 2:** Parameters assumed for the simulations.

Parameter	Value
Ambient temperature ($T_{0}$)	293 K
Ambient pressure ($p_{0}$)	1 bar
Temperature at LNG turbine inlet ($T_{e_{LNG}}$)	283 K
Temperature at ORC turbine inlet ($T_{e_{ORC}}$)	283 K
LNG pump adiabatic efficiency ($\eta_{p_{LNG}}$)	75%
ORC pump adiabatic efficiency ($\eta_{p_{ORC}}$)	75%
LNG expander adiabatic efficiency ($\eta_{e_{LNG}}$)	75%
ORC expander adiabatic efficiency ($\eta_{e_{ORC}}$)	75%
Minimal turbine outlet pressure	1.1 bar
Turbine outlet vapor fraction	1

**Table 3:** Parameters for each point of direct expansion cycle.

Point	*T* (K)	*p* (bar)	*h* (kJ/kg)	*s* (kJ/(kg·K))
1	109.1	1	−5452	4.626
2	112.5	63	−5433	4.684
3	283	63	−4656	8.856
4	136.4	1	−4899	9.526
5	293	1	−4557	11.23

**Table 4:** Parameters for each point of 2-stage direct expansion cycle.

Point	*T* (K)	*p* (bar)	*h* (kJ/kg)	*s* (kJ/(kg·K))
1	109.1	1	−5452	4.626
2	114	100	−5422	4.718
3	283	100	−4610	8.950
4	168	10	−4750	9.637
5	283	10	−4497	9.831
6	189.9	1	−4680	10.18
7	293	1	−4557	1.23

**Table 5:** ORC working fluids and their characteristic parameters.

Working Fluid	$T_{\mathrm{Boiling}}$ (K)	$p_{\mathrm{Critical}}$ (bar)	$T_{\mathrm{Critical}}$ (K)	$T_{\mathrm{Solidification}}$ (K)	GWP (-)	ODP (-)
Ethane	184.6	49	305.3	101	5.5	0
Methane	111.6	46	190.6	90.6	25	0
Ethylene	169.5	50.6	282.5	104	3.7	0
HFC-23	191.1	48.2	2993	118	14,800	0
PFC-14	145.3	37.5	277.5	89.5	7390	0

**Table 6:** Parameters for each point of ORC cycle.

Point	*T* (K)	*p* (bar)	*h* (kJ/kg)	*s* (kJ/(kg·K))	${\dot{m}}_{\mathrm{ORC}}/{\dot{m}}_{\mathrm{LNG}}$
1	109.1	1	−5452	4.626	1
2	133.3	1	−4890	9.591	1
I	121.5	1	−5046	9.541	1.05
II	111.4	1	−5580	4.757	1.05
III	115.7	79	−5555	4.83	1.05
IV	283	79	−4801	8.817	1.05

**Table 7:** Values of maximum exergetic efficiency and the corresponding optimal pumping pressures in the combined exergy recovery system.

Fluid	Exergetic Efficiency (%)	LNG Pump Pressure (Bar)	ORC Pump Pressure (Bar)
Ethane	36.2	21	36
Propane	30.8	34	8
HFC-23	34.1	32	20
PFC-14	34.3	15	63

**Table 8:** Parameters for each point of combined ORC-direct expansion cycle.

Point	*T* (K)	*p* (bar)	*h* (kJ/kg)	*s* (kJ/(kg·K))	$\dot{m}/{\dot{m}}_{\mathrm{LNG}}$
1	109.1	1	−5452	4.626	1
2	110.8	21	−5446	4.644	1
3	175.1	21	−4866	8.368	1
4	283	21	−4603	9.548	1
5	166.2	1	−4823	10.04	1
6	293	1	−4557	11.23	1
I	184.2	1	−3246	4.408	1.35
II	184.2	1	−3487	3.094	1.35
III	186.4	36	−3479	3.111	1.35
IV	283	36	−3194	4.315	1.35
